# Research on the pathogen causing root rot on *Phyllanthus emblica* from the perspectives of identification, bionomics, fungicide sensitivity assay, and transcriptome analysis under different pH stress

**DOI:** 10.3389/fmicb.2025.1612979

**Published:** 2025-07-31

**Authors:** Yiming Zhang, Minyan Song, Yonghong He, Yehua Si

**Affiliations:** ^1^School of Tourism Management, Huzhou Vocational and Technical College, Huzhou, China; ^2^College of Forestry and Biotechnology, Zhejiang Agricultural and Forestry University, Hangzhou, China; ^3^College of Plant Protection, Yunnan Agricultural University, Kunming, China; ^4^Tanya College, Hainan University, Danzhou, China

**Keywords:** crop protection, *Phyllanthus emblica*, root rot disease, *Diaporthe eres*, EC_50_

## Abstract

In recent years, *Phyllanthus emblica* root rot disease has occurred in Yunnan and caused significant economic losses to local plantation farmers, the pathogen was identified as *Diaporthe eres* through morphological and molecular method. The indoor toxicity testing and biological characteristic research on *Diaporthe eres* were carried out. Additionally, the transcriptional regulatory mechanism under different pH stress conditions was studied. The results showed that Prochloraz had the strongest inhibitory effect on *Diaporthe eres* mycelial growth, with an EC_50_ value of 0.059 mg·L-1. *Diaporthe eres* grows best in an environment of 26°C, 12: 12 alternating light, and pH 6. Go enrichment analysis revealed that the differentially expressed genes were mainly related to the biosynthesis of ribonucleoprotein complexes, ncRNA metabolic processes, ribosome and so on. KEGG analysis shows that in acidic environments, *Diaporthe eres* responds to external stress by precisely regulating amino acid metabolism and ribosome function, while in alkaline environments, it helps cells respond to and perceive external stress by forming multi-level adaptive networks and inhibiting activities such as protein synthesis transcription. This study provides relevant references for the prevention and control of this disease.

## 1 Introduction

*Phyllanthus emblica*, a deciduous tree of the Euphorbiaceae family, is well known for its nutrient-rich fruits, which contain high levels of vitamin C and various bioactive compounds, including flavonoids, polyphenols, and tannins (Sripanidkulchai and Junlatat, 2014). It has been used for both medicinal and dietary purposes in China for nearly 2,000 years. The tree can grow up to 23 meters in height with a trunk diameter of 50 cm (Luo et al., 2018; Yang et al., 2018). *P. emblica* is widely distributed across India, Sri Lanka, the Indochinese Peninsula, Indonesia, Malaysia, and southwestern China (Yang et al., 2008), with Yunnan Province being one of the primary regions for its cultivation. In recent years, *P. emblica* cultivation has increased rapidly in Yunnan due to strong farmer interest and promising market prospects. The species shows high adaptability in the Nujiang River Area, Jinsha River Area, and Lancang River Area of western Yunnan, where natural populations are abundant. It possesses traits such as drought resistance, tolerance to poor soils, rapid growth, early fruiting, and a long economic lifespan (Wu et al., 2016). Common diseases affecting *P. emblica* include fruit soft rot caused by *Penicillium choerospondiatis* (Wu et al., 2019), fruit anthracnose caused by *Colletotrichum gloeosporioides* (Priyamedha et al., 2020), black powder disease caused by *Alternaria* spp. (Zheng et al., 2014), stem trunk galls caused by *Splanchnonema Phyllanthus*, and fruit spots caused by *Diaporthe phoenicicola*. Among these, black powder disease has the highest incidence rate, affecting up to 72.4% of fruits. Surveys indicate that over 98% of fallen fruits show disease symptoms, and 13–15% of unfallen fruits are also affected. While research on *P. emblica* diseases has primarily focused on fruit, branches, and leaves, studies on root diseases remain limited. In recent years, root rot in *P. emblica* has become increasingly prevalent in Yunnan Province. Due to a lack of research and management strategies, farmers have been unable to implement effective fungicide treatments, leading to significant yield reductions and economic losses. Currently, no studies have identified the causal pathogen of *P. emblica* root rot or assessed its biological characteristics and fungicide sensitivity. This study focus on identifying the root rot pathogen through morphological analysis and DNA-based molecular identification, as well as to investigate its biological characteristics to determine optimal growth conditions. Furthermore, the *in vitro* toxicity of six fungicides against *Diaporthe eres* was evaluated to provide a foundation for future field trials and more effective disease management strategies.

The pH value significantly affects fungal growth, as an appropriate pH is essential for microbial survival. Environmental pH fluctuations present major challenges to microorganisms, as acid–base stress can disrupt intracellular pH homeostasis, thus affecting the structure and function of biological macromolecules such as proteins, nucleic acids, lipids, and carbohydrates (Krulwich et al., 2011; Gupta et al., 2020). When external pH deviates from the optimal range, microorganisms need one or more regulatory pathways to maintain intracellular pH near neutral, ensuring normal growth and cellular function (Guo et al., 2019; Mamo, 2020). Pathogens adapt to environmental changes during host-pathogen interactions through metabolic adjustments and gene expression regulation to support survival and reproduction (Yin and Keller, 2011; Dong and Ma, 2021; Jiang et al., 2021). Research on plant diseases primarily focuses on the pathogenic processes of pathogens and the host’s gene expression response to infection. Most studies have investigated the infection mechanisms and pathogenicity of *Diaporthe eres*, along with the gene expression regulation of host responses (Boenisch and Schäfer, 2011; Fernandes et al., 2017). However, limited attention has been given to gene expression changes within *Diaporthe eres* itself. Therefore, this study systematically explores the gene expression pathways and physiological mechanisms of *Diaporthe eres* under different pH conditions, providing valuable insights into its regulatory responses to pH variations.

## 2 Materials and methods

### 2.1 Samples collection

**Figure 1:**
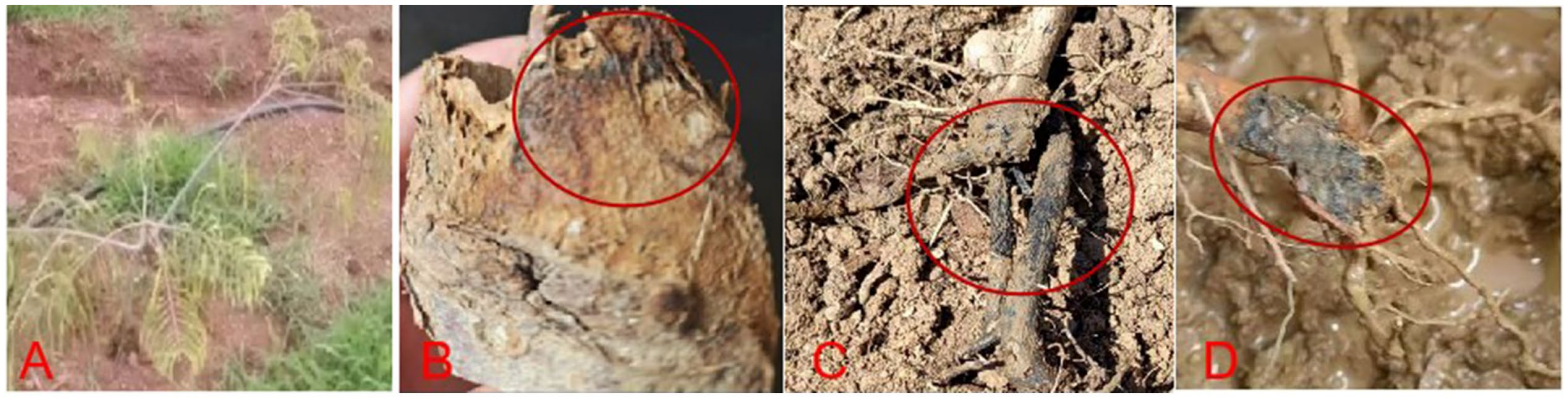
Rot root disease on *P. emblica.*

**Figure 2:**
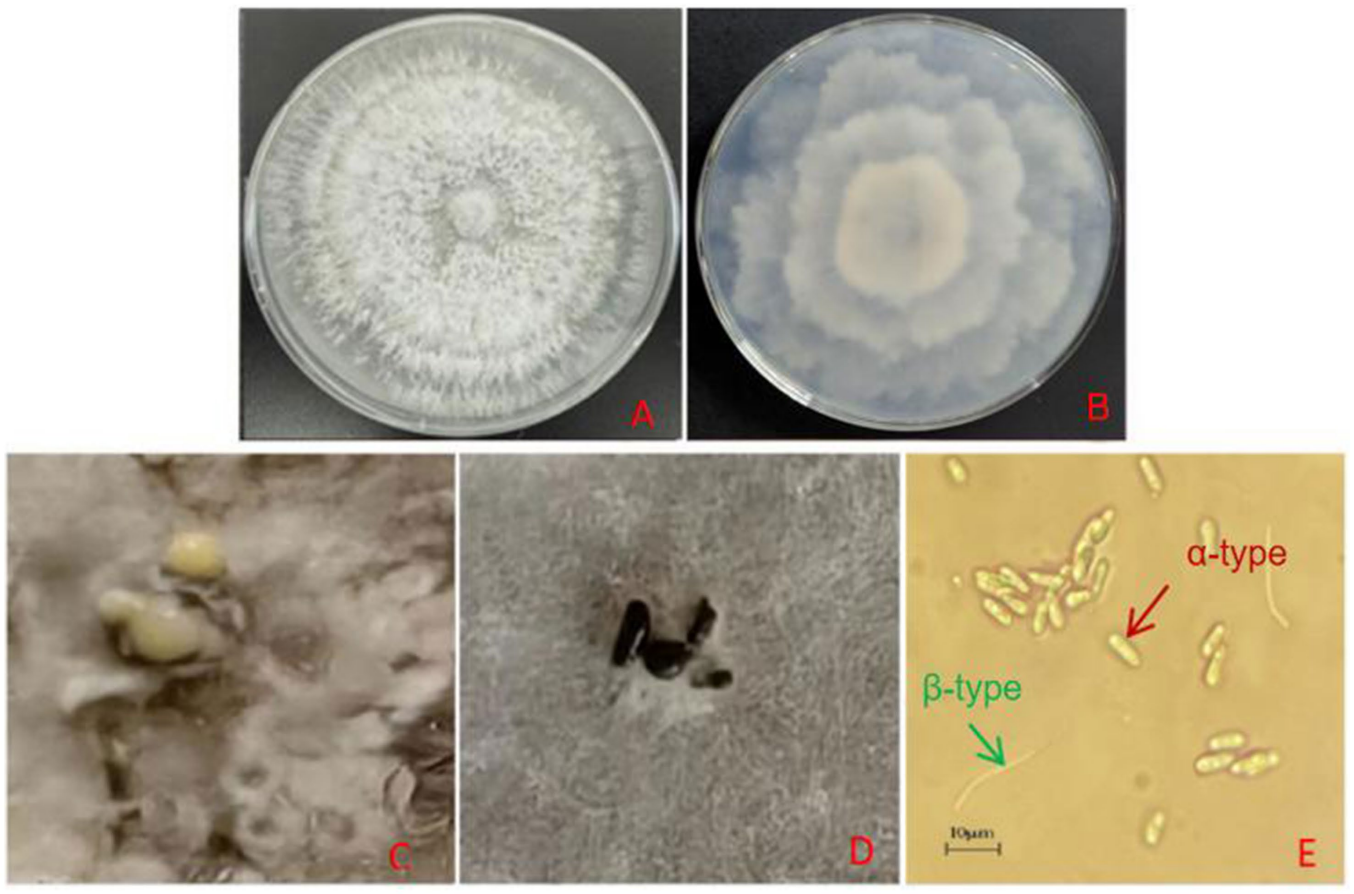
**(A)** Colony morphology on PDA (front view, 10 days). **(B)** Colony morphology on PDA (reverse view, 10 days). **(C)** Conidial angle (65 days). **(D)** Black conidiomata formation on the colony (35 days). **(E)** *α*-conidia and *β*-conidia (58 days).

**Figure 3:**
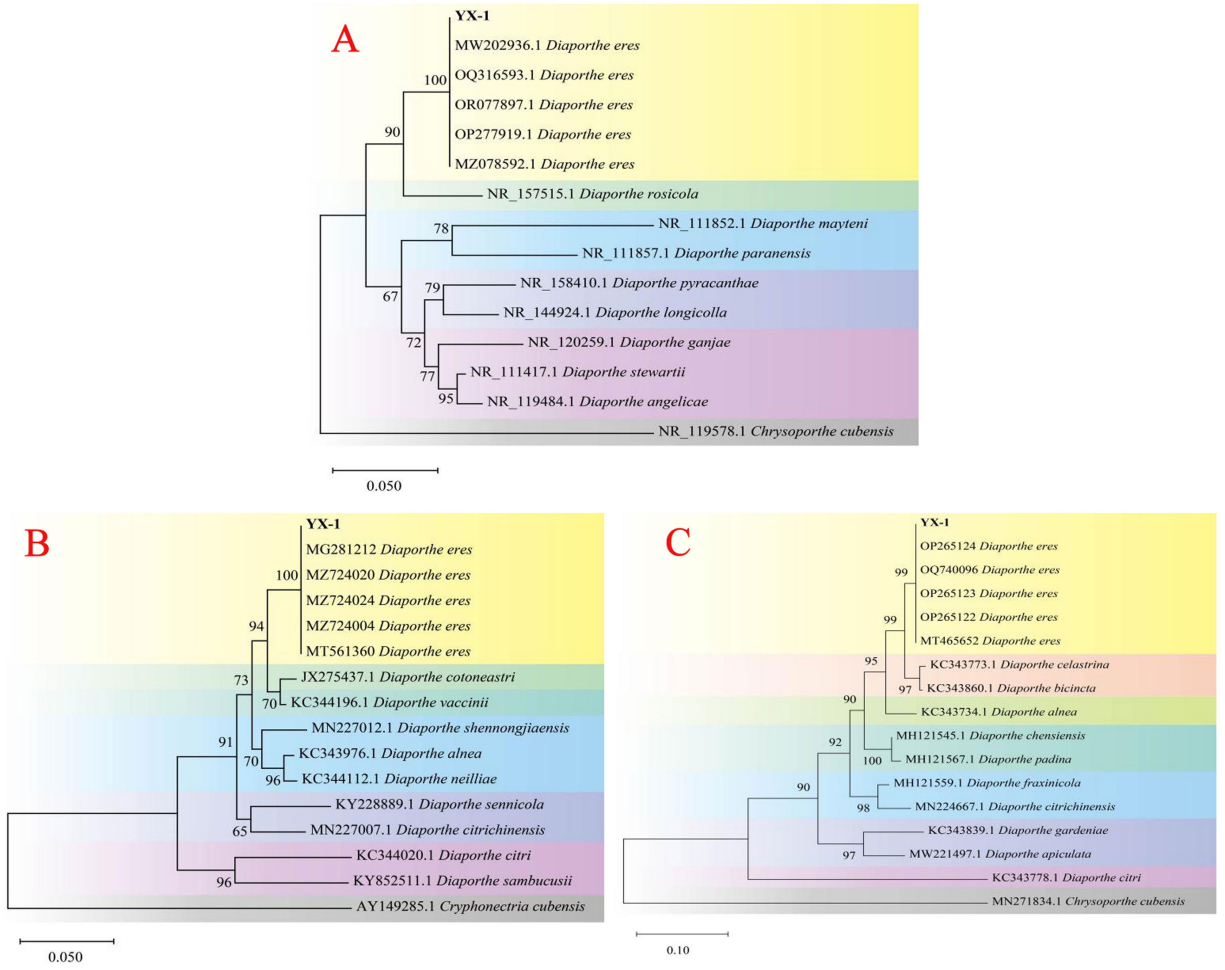
Phylogenetic tree of YX-1 strain based on ITS sequences **(A)**, EF*1-α* sequences **(B)** and *TUB2* sequences **(C)**.

In March 2024, samples with typical disease characteristics were collected from a *P. emblica* farm in Guojiaying Community, Ningzhou Sub-district, Hua County, Yuxi City, Yunnan Province (E: 102°49′, N: 24°34′). Compared with healthy plants, diseased *P. emblica* had the following symptoms: (1) early-stage plants were stunted and wilted (Figure 1A); (2) in the middle stage, root cortex decay was observed, with brown to dark brown lesions forming (Figures 1B,C); (3) in the advanced stage, affected plants fell over and died (Figures 1C,D).

### 2.2 Isolation and purification of pathogen

The pathogen was isolated using the tissue isolation method (Cao et al., 2018). A 1 cm × 1 cm tissue block was excised from the junction of diseased and healthy root tissue of *P. emblica*. The samples were immersed in 75% ethanol for 30 s for surface disinfection, rinsed with sterile water 3–5 times, then soaked in 2% sodium hypochlorite for 1 min, and rinsed with sterile water again 3–5 times. Before transferring the tissue onto potato dextrose agar (PDA) medium, the excess moisture was removed by sterile filter paper. Cultures were incubated at 25°C for 5–7 days. Growing mycelia were picked with a needle in a sterile laminar flow hood, transferred aseptically to fresh PDA medium, and subcultured for another 5–7 days. Single-spore isolation and purification were performed using the gradient dilution method until a pure pathogen culture was obtained (Leslie and Summerell, 2006). The purified isolates were stored at 4°C for further use.

### 2.3 Pathogenicity testing

The surface of 3-year-old healthy *Phyllanthus emblica* roots was disinfected with 75% ethanol for 30 s and then rinsed three times with sterile water. Pathogenicity testing for isolated and purified strains was conducted using the needle puncture method (Gupta et al., 2020). Small wounds were made on the roots using a sterile anatomical needle, and a 0.5 cm mycelial plug was placed onto each wound with the mycelial side facing down. Sterile PDA plugs served as controls, with three replicates per group. After inoculation, the roots were wrapped in cling film and monitored for 15 days. Infected roots were then dissected to observe internal tissue colonization. Moreover, longitudinally sectioned healthy *P. emblica* roots were inoculated with mycelial plugs for moist culture, and symptoms were observed over 4–15 days.

For pot-based inoculation, three-year-old healthy *P. emblica* plants were selected. Root surfaces were disinfected, and wounds were made on the primary and fibrous roots using a sterile inoculation needle. A prepared spore suspension (1 × 10^7^ spores/mL) was applied to the wounds, while control plants received sterile water. Inoculated plants were transplanted into sterile soil pots, with three replicates per group. Watering was done every 3 days. After 30 days, symptomatic tissues from inoculated plants were subjected to pathogen re-isolation and culture. The identity of the pathogen was confirmed based on Koch’s postulates (Xi et al., 2018).

### 2.4 Morphological identification of pathogen

After single spore isolation, the purified strain was cultured on PDA plates for 6 days under dark conditions at 25°C. Colony morphology, including color, size, and shape, was recorded. Once spores were produced, the morphological characteristics of conidia were examined using the microscope and the length and width of at least 30 conidia were measured (An et al., 2020; Zhang and Cao, 2022). The taxonomic status of the pathogen was preliminarily determined based on morphological characteristics and reference data.

### 2.5 Molecular identification of pathogen

CTAB method (Udayanga et al., 2012) was used for extracting the Genomic DNA and the sequences of the pathogenic were amplified using *ITS*, EF1-*α*, and *TUB2* primers. All primers (Supplementary Table S1) were synthesized by Youkang Biotech (Hangzhou) Co., Ltd. Polymerase chain reaction (PCR) was performed in a 50 μL reaction system consisting of 25 μL 2 × Taq PCR Master Mix, 21 μL ddH₂O, 1 μL of each 10 μmol/L primer, and 2 μL template DNA. The reaction conditions were as follows: initial denaturation at 95°C for 3 min, 34 cycles of 95°C for 30 s, annealing at the corresponding temperature for 30 s, and extension at 72°C for 1 min, followed by a final extension at 72°C for 10 min (Zhou et al., 2022). PCR products were analyzed by electrophoresis and sent to Youkang Biotech (Hangzhou) Co., Ltd. for sequencing. The obtained sequences were compared using the NCBI database, and the sequences were submitted. Neighbor-joining (NJ) method was used to construct a phylogenetic tree in MEGA 7.0 software.

### 2.6 *In vitro* toxicity test of six fungicides against *Diaporthe eres*

The *in vitro* toxicity test was conducted using the mycelial growth rate method (Mu, 1994). PDA plates containing different concentrations of the six fungicides (Supplementary Table S2) were prepared, while PDA plates supplemented with an equal volume of sterile water served as the blank control. Each treatment was performed in triplicate. Mycelial plugs (*Φ* = 5 mm) were excised using a puncher and inoculated onto PDA plates containing the various fungicide concentrations and the control. The plates were incubated in a constant-temperature incubator at 25°C for 6 days. Colony diameters were measured using the cross-method, and the mycelial growth inhibition rate was calculated.

The inhibition rate was converted into the corresponding probability value using a biological statistics probability conversion table. The probability value was plotted as the vertical coordinate, and the logarithm of each fungicide concentration was plotted as the horizontal coordinate to determine the toxicity regression equation. The inhibition rate, EC_50_ value, slope, and correlation coefficient (*r*) for each fungicide were calculated (Blanco et al., 2008).

### 2.7 Research on the bionomics of the pathogen

A mycelial plug (*Φ* = 5 mm) of the tested strain was inoculated at the center of a PDA plate and incubated at 25°C under three light conditions: continuous light (24 h light),12:12 light–dark cycle, and 24 h dark for 5 days. Each treatment was performed in triplicate. To assess the effect of different culture media, a mycelial plug (*Φ* = 5 mm) was inoculated onto PDA (20 g agar, 200 g peeled potato, 20 g glucose, 1,000 mL sterile water), PSA (20 g agar, 20 g sucrose, 200 g peeled potato, 1,000 mL sterile water), OMA (20 g agar, 30 g oatmeal, 1,000 mL sterile water), CMA (20 g agar, 40 g corn flour, 1,000 mL sterile water), CPK (1 g dipotassium hydrogen phosphate, 20 g agar, 2 g sodium nitrate, 0.5 g potassium chloride, 0.01 g ferrous sulfate heptahydrate, 20 g sucrose, 0.5 g magnesium sulfate heptahydrate, 1,000 mL sterile water), RD (20 g agar, 200 g fresh *P. emblica* root stock, 20 g glucose, 1,000 mL sterile water), and WA (20 g agar, 1,000 mL sterile water) media and incubated in the dark at 25°C for 5 days, with three replicates per treatment. PDA medium was adjusted to pH 3–11 using 1 mol/L HCl or 1 mol/L NaOH for pH tolerance analysis. A mycelial plug (*Φ* = 5 mm) was inoculated at the center of each adjusted medium and incubated in the dark at 25°C for 5 days, with three replicates per treatment. To examine the effect of temperature, mycelial plugs were inoculated onto the PDA medium and incubated in the dark at 5, 10, 15, 20, 25, 30, and 35°C for 5 days with three replicates per treatment. For nitrogen and carbon source utilization studies, modified Czapek (CPK) media were prepared by replacing sodium nitrate with glycine, beef extract, urea, peptone, potassium nitrate, or ammonium sulfate in equal amounts. Similarly, carbon sources were varied by replacing sucrose with soluble starch, D-fructose, glucose, maltose, or D-galactose in equal amounts. Media without nitrogen or carbon sources served as blank controls. Mycelial plugs (*Φ* = 5 mm) were inoculated at the center of each test medium (one plug per dish) and incubated in the dark at 25°C for 5 days, with three replicates per treatment. Colony diameters were measured using the cross-method to determine growth differences under different conditions.

### 2.8 Data analysis

The mean diameter of colonies, inhibition rates, EC50 values, correlation coefficients, significant differences and regression equations were calculated using SPSS 19.0 (SPSS Inc., Chicago, Illinois, USA). Three replicates were set for each experimental treatment. Data are expressed as mean ± standard deviation. Differences between groups were analyzed with one-way ANOVA. The significant difference was defined at *p* < 0.05.

### 2.9 Strain cultivation and RNA extraction

*Diaporthe eres* was inoculated onto PDA medium and cultured at 28°C for 7 days. A sterile pipette tip was used to punch out mycelial plugs from the edge of the colony and 10 plugs (Φ = 20 mm) were transferred into the PDB medium (150 mL). After culturing in a shaker at 28°C (150 r/min) for 4 days (Yu et al., 2021), fresh mycelium (1.0 g for each treatment) was collected using 4 layers of gauze and transferred to PDB liquid medium (150 mL) with pH values of 4.5, 6.5 and 8.0, respectively.

The mycelium were incubated under constant temperature conditions at 28°C with shaking at 200 r/min in the dark for 2 days (Shang et al., 2024) and were washed with sterile water, then rapidly frozen in liquid nitrogen (Zhang et al., 2022). There were three biological replicates for each treatment and all collected samples were stored at −80°C before being sent to Nanjing Xianyuan Biotechnology Co., Ltd. for RNA extraction, RNA library construction, and sequencing.

### 2.10 Transcriptome data processing

Fastp software was used to assess the quality of the sequencing data (Dissanayake et al., 2015), and low-quality reads were filtered out. The clean reads were aligned to the reference genome of *Diaporthe eres*^1^ with accession number ASM2257077v2 using HISAT2.

### 2.11 GO and KEGG enrichment analysis of differentially expressed genes

FeatureCounts software was used to quantify clean reads. Differential expression analysis of gene transcription levels among the three pH-treated samples was conducted using DESeq2. Genes with *p* < 0.05 and |log₂ fold change| > 1 were considered differentially expressed (DEGs). Gene Ontology (GO) enrichment analysis and Kyoto Encyclopedia of Genes and Genomes (KEGG) enrichment analysis of DEGs were performed using clusterProfiler (Kanehisa and Goto, 2000; Kanehisa, 2019; Aramaki et al., 2020; Kanehisa et al., 2023).

### 2.12 Quantitative real-time PCR analysis

To further verify the reliability of the RNA-seq experimental results, the total RNA of *Diaporthe eres* mycelia from the experimental groups (pH = 4.5/pH = 8.0) and the control group (pH = 6.5) was extracted. The RNA was reverse transcribed into cDNA using a reverse transcription kit. Six DEGs were selected, and gene-specific primers were designed based on the gene sequences using Primer Premier 5 software. The primers were synthesized by Youkang Biotechnology (Hangzhou) Co., Ltd. The primer names and sequences are shown in Supplementary Table S3. After the primers were verified to be qualified, RT-qPCR analysis was performed using the Hieff@ qPCR SYBR Green Master Mix fluorescence quantitative premix kit (Yisheng Biotechnology Co., Ltd.). *Tubulin* was used as the internal reference gene, and the 2^−ΔΔCt^ (Livak and Schmittgen, 2001) was adopted for relative quantification.

## 3 Results

### 3.1 Morphological identification of the pathogen

After 10 days of culture on PDA medium, the mycelium was dense and milky white with an irregular edge (Figures 2A,B). After 35 days in the PDA medium, black-brown conidiomata with irregular shapes and papillary tops were scattered on the colony surface (Figure 2D). After 65 days, yellow conidial sterigmata were observed (Figure 2C). Microscopic examination of 58-day-old cultures revealed both *α*-type and *β*-type conidia (Figure 2E). The α-type conidia were single-celled, transparent, colorless, and ellipsoidal to fusiform, with a distinct oil drop at each end, measuring 6.5–8.9 μm × 2.4–3.1 μm, with an average size of 7.99 μm × 2.84 μm (*n* = 30). The β-type conidia were single-celled, transparent, colorless, linear, often hooked at one end, and without oil drops, measuring 21.2–32.4 μm × 1.29–1.84 μm. Based on the above observation results, we have preliminarily identified the pathogen as *Diaporthe* spp., which belong to Ascomycota, Sordariomycetes, Diaporthales, and Diaporthaceae. The asexual stage was classified as *Phomopsis* spp.

### 3.2 Molecular identification of the pathogen

The genomic DNA of strain YX-1 was amplified using ITS1/ITS4, EF1-728F/EF1-986R, and Bt-2a/Bt-2b primer pairs, resulting in sequences of 558 bp, 330 bp, and 416 bp, respectively. The above sequencing results have been uploaded to the NCBI database with accession numbers PV170938, PV177466, and PV177465. A phylogenetic tree based on the *ITS* gene sequence (Figure 3A) showed that strain YX-1 clustered with *Diaporthe eres* isolates (registration numbers MW202936.1, OQ316593.1, OR077897.1, OP277919.1, and MZ078592.1) with a support rate of 100%. A second phylogenetic tree based on the EF1-α gene sequence (Figure 3B) revealed that strain YX-1 shared 100% sequence similarity with *Diaporthe eres* isolates (registration numbers OP265124, OQ700096.1, OP265123.1, OP265122.1, and MT465652.1). A third phylogenetic tree based on the *TUB2* gene sequence (Figure 3C) indicated 99% sequence similarity between strain YX-1 and *Diaporthe eres* isolates (registration numbers MG281212, MZ724020, MZ724024, MZ724000, and MT561360). Based on the combined molecular analyses and morphological characteristics, strain YX-1 was identified as *Diaporthe eres*, with its asexual stage classified as *Phomopsis eres*. The sexual state belongs to Ascomycota, Pyrenomycetes, Sphaeriales, and *Diaporthe*, while the asexual state belongs to Imperfecti, Coelomycetes, Sphacropsis, and *Phomopsis*.

### 3.3 Incidence and symptoms of *Diaporthe eres*

**Figure 4:**
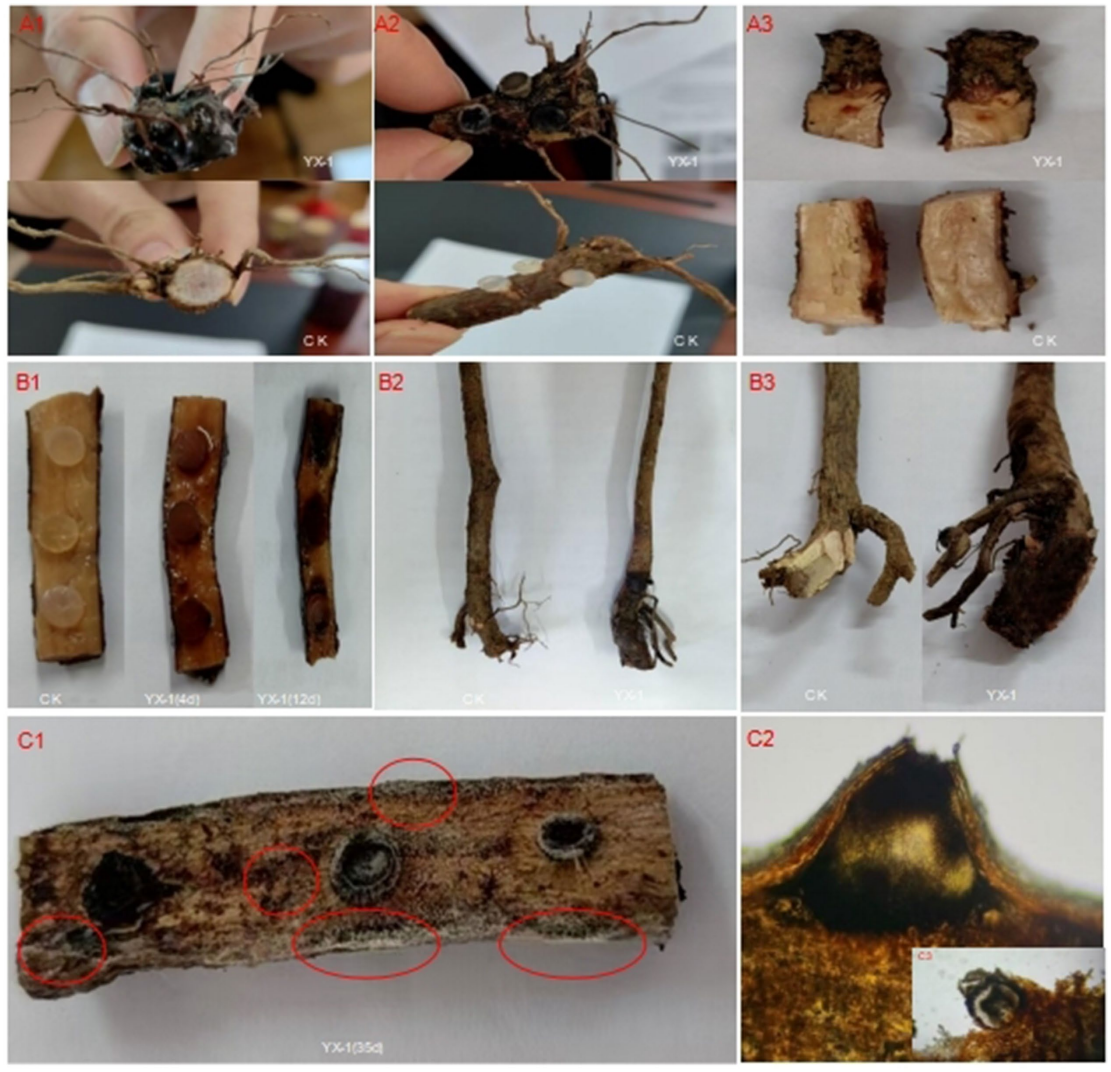
Pathogenicity test of *P. emblica* root rot. **(A1–A3)** Disease susceptibility observed 15 days after inoculation with pathogenic fungi. **(B1)** Slitting phenotype after 4 and 12 days of plug inoculation. **(B2,B3)** Root surface and sectional symptoms observed in pot anatomy after 30 days. **(C1)** Black conidiomata on tissues **(C2)** Morphology of *Diaporthe eres’* conidiomata.

*In vitro* inoculation experiments demonstrated that severe disease symptoms were observed 15 days after the inoculation of *P. emblica* roots with strain YX-1. The inoculated root surfaces turned black and rotted, with some of the fibrous roots covered by mycelia (Figure 4A2). Cross-sectional observation of infected roots revealed extensive tissue necrosis, whereas no symptoms were observed in the control group (Figure 4A1). Longitudinal sections of inoculated roots showed vigorous mycelium growth within the tissues (Figure 4A3). Compared with the control group, blackening of the tissue was observed in the main root as early as the fourth day after inoculation (Figure 4B1), with disease severity increasing by day 12. On the 20th day, the roots surface was covered with hyphae, and black conidiomata appeared on the 35th day (Figure 4C1). After microscopic examination of conidiomata, Acervulus of *Diaporthe eres* were also found (Figures 4C2,C3).

In pot inoculation experiments, 30 days after pathogen inoculation, *P. emblica* roots exhibited symptoms similar to those observed in the field, whereas control plants remained healthy. The infected roots turned black and rotted, with black spots covering the surface (Figures 4B2,B3), while control plants showed normal growth.

Re-isolation and culturing of the pathogen from diseased tissues yielded isolates with morphological characteristics identical to those of the inoculated strain, confirming *Diaporthe eres* as the causal agent of root rot in *P. emblica*.

### 3.4 Effects of six different fungicides on the growth of *Diaporthe eres*

The six tested fungicides showed varying degrees of inhibitory effects on the mycelial growth of *Diaporthe eres* at different concentrations. The inhibition rates for Tebuconazole (97.3%), Difenoconazole (95%), Prochloraz (95%), Carbendazim (95%), Azoxystrobin (98.5%), and Chlorothalonil (98%) ranged from 5.23 to 87.36%, 10.66 to 83.66%, 5.15 to 86.40%, 8.30 to 85.12%, 5.23 to 87.36%, and 8.10 to 86.27% (Supplementary Table S4), respectively. Prochloraz (95%) had the strongest inhibitory effect on *Diaporthe eres*, with an EC50 value of 0.059 μg·mL^−1^, followed by Difenoconazole (95%), which had an EC50 value of 0.128 μg·mL^−1^ (Supplementary Table S5). Carbendazim (95%) and Tebuconazole (97.3%) also effectively inhibited mycelial growth, with EC50 values of 0.616 μg·mL^−1^ and 1.063 μg·mL^−1^, respectively. On the other hand, Azoxystrobin (98.5%) had the weakest inhibitory effect, with an EC50 value of 32.226 μg·mL^−1^.

### 3.5 Bionomics of *Diaporthe eres*

**Figure 5:**
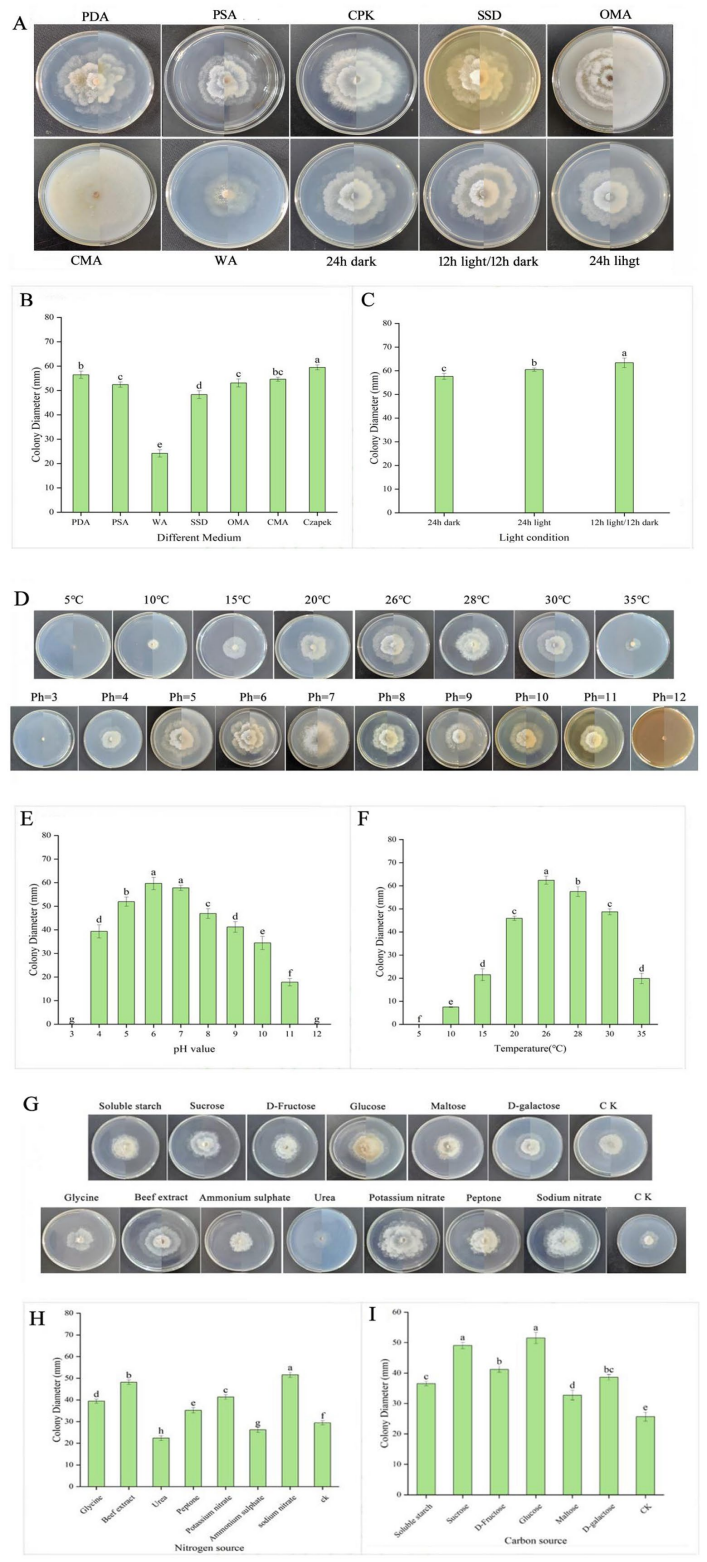
The mycelial growth diameters under different medium and light conditions **(A–C)**, temperatures and pH values **(D–F)** and carbon and nitrogen sources **(G–I)**.

*Diaporthe eres* was able to grow on all tested media. A yellow pigment appeared on the mycelium within 5 days on PDA and OMA media. Mycelial growth was fastest on CPK medium, with a colony diameter of 59.4 mm, followed by PDA with a diameter of 56.4 mm. Growth was slowest on WA medium, with a diameter of 24.2 mm (Figures 5A,B). The optimal light condition for *Diaporthe eres* was 12 h light/12 h dark, where the colony diameter reached 63.4 mm, significantly larger than those under continuous light or darkness (Figures 5A,C).

*Diaporthe eres* had normal growth at temperatures ranging from 10°C to 30°C, with the fastest growth observed at 26°C, where the average colony diameter was 62.4 mm. At 15 and 20°C, the colony diameters were 21.5 and 45.6 mm, respectively. No signs of growth were observed on *Diaporthe eres* at both 5 and 35°C (Figures 5D,F).

The mycelium of *Diaporthe eres* grew on the PDA medium with pH values ranging from 4 to 11, with optimal growth at pH 5 to 7. The largest colony diameter (59.7 mm) was observed at pH 6 on the 5^th^ day of culture, indicating that a weakly acidic environment favors *Diaporthe eres* growth. Therefore, adjusting soil pH could potentially reduce disease occurrence. For instance, applying plant ash could not only supplement nutrients but also provide a preventive effect by slightly modifying soil pH. The mycelium growth ceased at pH 3 and 12 (Figures 5D,E).

Among the nitrogen sources, sodium nitrate had the most significant effect, with a colony diameter of 51.6 mm. The colony diameters in media supplemented with glycine, beef extract, ammonium sulfate, peptone, and potassium nitrate were 39.4 mm, 48.2 mm, 26.2 mm, 35.2 mm, and 41.4 mm, respectively. The weakest promoting effect was observed in the urea-supplemented medium, where the colony diameter was 22.4 mm (Figures 5G,H). Thus, sodium nitrate was the most effective nitrogen source for *Diaporthe eres*.

Glucose had the most significant effect on the growth of *Diaporthe eres* among the carbon sources, with a colony diameter of 51.5 mm (Figures 5G,I). Sucrose and D-fructose also promoted growth, with colony diameters of 49.1 and 41.2 mm, respectively. Maltose, soluble starch, and D-galactose had the weakest effects, with colony diameters of 32.7 mm, 36.5 mm, and 38.6 mm, respectively. Therefore, glucose was determined to be the best carbon source for *Diaporthe eres.*

### 3.6 Transcriptome data quality assessment

An overview of the sequencing and mapping results were listed in Supplementary Table S6. Approximately 7,347,954–11,652,356 clean reads per processing were obtained. After mapping clean reads to the *Diaporthe eres* genome, approximately 84.21–93.45% reads were successfully aligned. The quality assessment of transcriptome data from three biological replicates showed that Q20 and Q30 values exceeded 98.76 and 95.90%, respectively, with G + C content ranging from 57.24 to 57.72% (Supplementary Table S6). These results indicate that the sequencing data are of high quality and suitable for subsequent transcriptome analysis.

**Figure 6:**
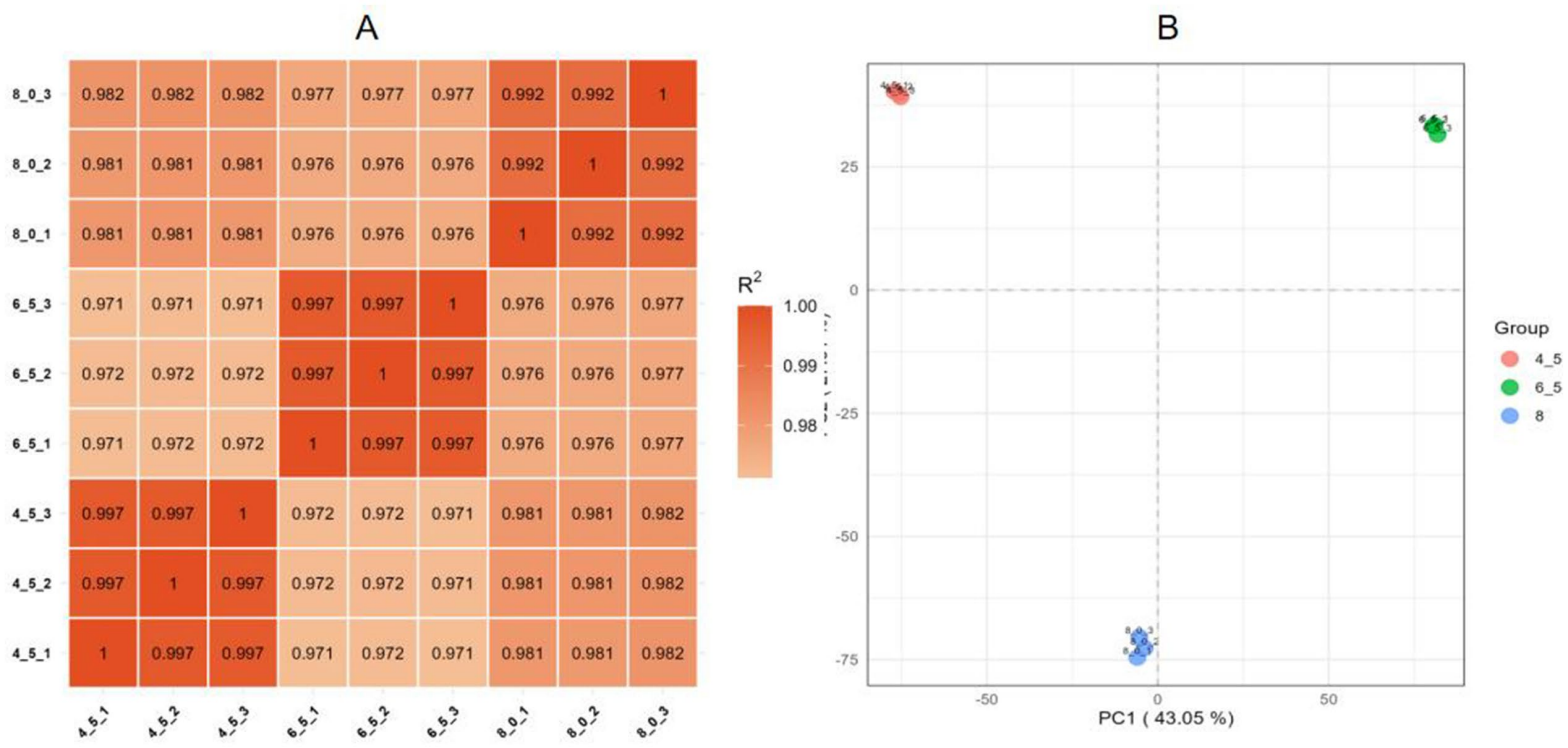
Reproducibility of *Diaporthe eres* transcriptome data under different pH treatments. **(A)** Heatmap of RNA expression values across different samples, with Pearson correlation coefficients represented by color and numerical values. **(B)** Principal component analysis (PCA) showing the distances between samples from different treatment groups.

Pearson correlation analysis was performed to evaluate the consistency of biological replicates, revealing correlation coefficients ranging from 0.972 to 0.997, confirming strong reproducibility among the samples (Figure 6A). Principal component analysis (Figure 6B) further demonstrated that all sample data were consistent and reliable for further analyses.

### 3.7 Analysis of the number of upregulated and downregulated genes

**Figure 7:**
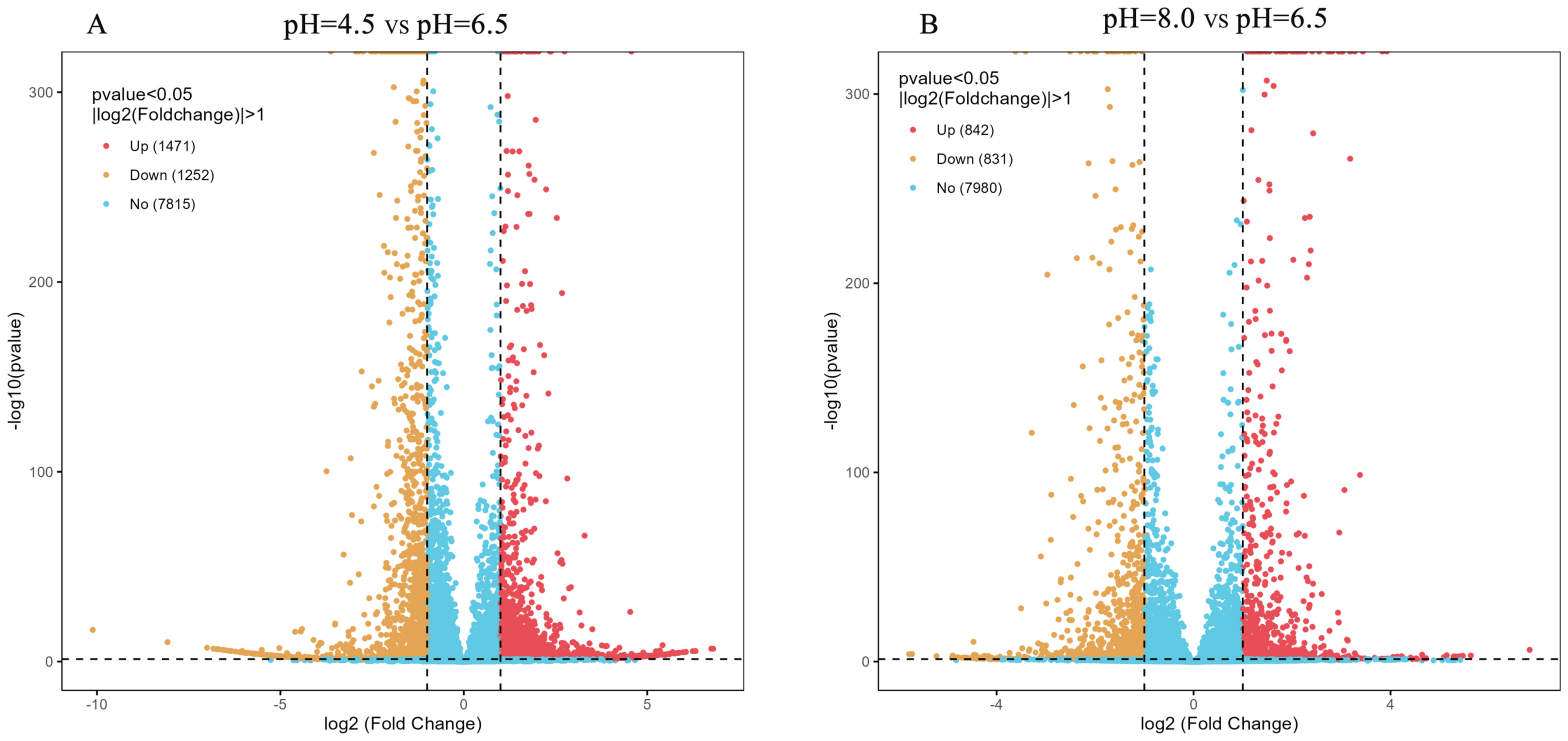
Volcano map of DEGs on *Diaporthe eres* under acidic and alkaline conditions.

Differential gene expression analysis was performed on three samples treated with different pH levels, with *p* < 0.05 and |log₂FC| > 1 considered as differentially expressed genes (DEGs). Compared with the control group (pH 6.5), 2,733 DEGs were identified at pH 4.5, including 1,471 upregulated and 1,252 downregulated genes (Figure 7A). At pH 8.0, 1,673 DEGs were detected, with 842 upregulated and 831 downregulated genes (Figure 7B).

### 3.8 GO enrichment analysis and KEGG enrichment analysis of differential expression genes

**Figure 8:**
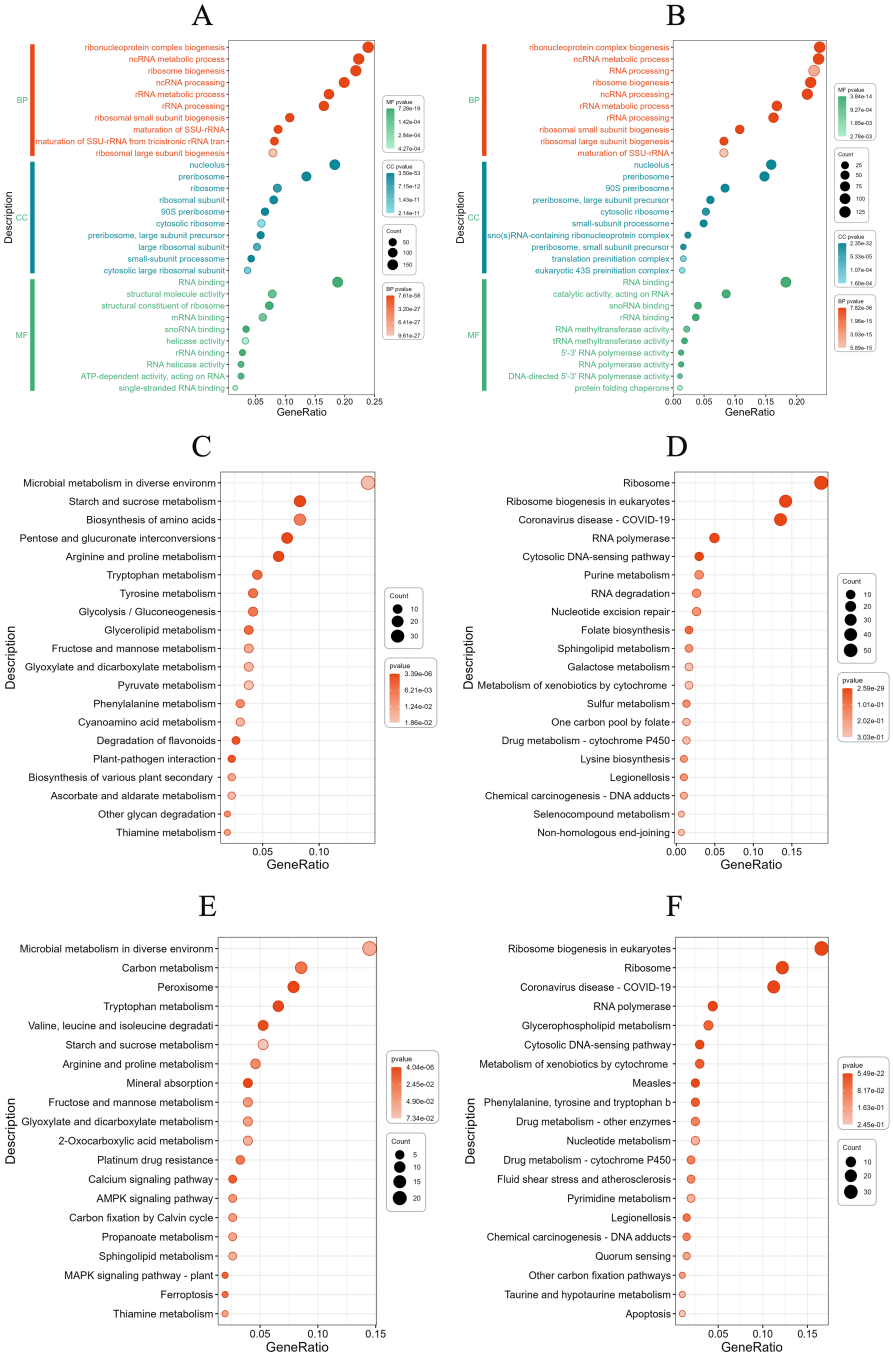
**(A)** Gene Ontology (GO) enrichment analysis of differentially expressed genes (DEGs) under acidic stress. **(B)** GO enrichment analysis of DEGs under alkaline stress. **(C)** KEGG enrichment analysis of up-regulated DEGs at acid stress. **(D)** KEGG enrichment analysis of down-regulated DEGs at acid stress. **(E)** KEGG enrichment analysis of up-regulated DEGs at alkaline stress. **(F)** KEGG enrichment analysis of down-regulated DEGs at alkaline stress.

GO enrichment analysis revealed that under pH 4.5 conditions, DEGs were primarily associated with ribonucleoprotein complex biogenesis, ncRNA metabolic processing, and ribosome biogenesis in biological processes. In cellular components, DEGs were mainly enriched in the nucleolus, preribosomes, and ribosomes. Regarding molecular function, the most enriched categories included RNA binding, structural molecular activity, and structural constituent of ribosome (Figure 8A). Under pH 8.0 conditions, DEGs were enriched in biological processes and cellular components similar to those observed under acidic conditions. In terms of molecular function, DEGs were primarily involved in RNA binding, catalytic activity, acting on RNA, and snRNA binding (Figure 8B). These findings suggest that *Diaporthe eres* adapts to intracellular and extracellular environmental changes by regulating ribonucleo protein complex production and modifying cellular metabolism under acid–base stress.

To further investigate the metabolic pathways influenced by DEGs under different pH conditions, KEGG enrichment analysis was performed. The results show KEGG pathway enriched by upregulating DEGs showed significant enrichment in the amino acid metabolism pathway under acidic conditions (Figure 8C). For example, the pathways of arginine and proline metabolism, tryptophan metabolism, and tyrosine metabolism indicates that cells may respond to stress by regulating amino acid metabolism. The genes SLS64_004997 (Glutamate 5-kinase) and SLS64_009143 (Proline dehydrogenase) are involved in proline synthesis and degradation, respectively, the gene SLS64_002201 (3-hydroxyanthranilic acid dioxygenase) catalyzes the conversion of 3-hydroxyanthranilic acid to 2-amino-3-carboxy glucuronic acid semialdehyde in the tryptophan metabolism pathway. This reaction is closely related to NAD + synthesis and may provide energy and coenzyme support for cells. The gene SLS64_002335 (succinate semialdehyde dehydrogenase) catalyzes the conversion of succinic acid semialdehyde into succinic acid, which enters the TCA cycle production capacity. The synergistic upregulation of these genes indicates that under acid stress, cells may maintain redox balance by activating amino acid catabolic pathways and providing precursor substances for important biosynthetic processes.

The ribosome pathway showed significant downregulation (Figure 8D), with SLS64_000533 (60 S ribosomal protein L3), SLS64_002840 (60S ribosomal protein L2), SLS64_003134 (40S ribosomal protein S12) and SLS64_005120 (54S ribosomal protein L7), SLS64_002938 (54S ribosomal protein L23), etc. are generally inhibited under acidic stress conditions. This global downregulation of ribosomal gene expression is closely related to the metabolic reprogramming of cells under stress conditions, manifested as: (1) reducing energy consumption by inhibiting protein synthesis; (2) synergistic downregulation of the ribosome biosynthesis regulatory factor ATPase activating ribosomal biosynthesis protein (SLS64_002972); (3) forming a regulatory network for metabolic resource redistribution with the significantly upregulated amino acid metabolism pathways (proline, tryptophan, and tyrosine metabolism) mentioned earlier. These changes collectively reflect the molecular mechanism by which cells achieve survival adaptation by finely regulating ribosome function in acidic environments.

Under alkaline stress conditions, cells form a systemic adaptation mechanism by synergistically regulating multiple metabolic pathways under alkaline stress conditions (Figure 8E). The significant upregulation of SLS64_012946 and SLS64_004253 (high affinity copper transporters) in the mineral absorption pathway indicates that cells maintain ion homeostasis by enhancing copper ion absorption; The activation of the peroxisome pathway is manifested by the synergistic upregulation of SLS64_000592 and SLS64_003452 (fatty acid metabolism related enzymes) and SLS64_008838 (antioxidant enzymes), enhancing the ability of fatty acid *β*-oxidation and reactive oxygen species scavenging; At the same time, the expression of SLS64_010853 (2-ketoglutarate dehydrogenase) and SLS64_010471 (aromatic transaminase) in the tryptophan metabolism pathway increased, promoting NAD + synthesis and redox balance. The synergistic regulation of these three pathways forms a multi-level adaptive network from ion balance, energy metabolism to oxidative defense, jointly helping cells cope with multiple stresses caused by alkaline environments.

Cells exhibit significant synergistic downregulation of ribosome synthesis and transcription machinery related genes under alkaline stress conditions (Figure 8F). Structural protein genes of 40S and 60S ribosomal subunits in the ribosomal pathway such as 60S ribosomal protein L3 (SLS64_000533) 60S ribosomal protein L2 (SLS64_002840),40S ribosomal protein S12 (SLS64_003134). At the same time, the ribosome biosynthesis regulatory factor ATPase activating ribosome biosynthesis protein (SLS64_002972) was significantly downregulated. Inhibition of ribosomal gene expression and RNA polymerase complex subunit genes such as DNA-directed RNA polymerase I subunit RPA2 (SLS64_001862), DNA-directed RNA polymerase III subunit C1 (SLS64_006317). The downregulation of protein synthesis and transcriptional activity in alkaline environments reflects the basic metabolic regulation strategy of cells to reduce energy consumption by inhibiting protein synthesis and transcriptional activity. It is worth noting that the expression of key enzyme genes, such as SLS64_011427 (3-deoxy-7-phosphoheptulonate synthase), SLS64_014097 (bifunctional chorismate synthase/riboflavin reductase) in the biosynthesis pathways of phenylalanine, tyrosine, and tryptophan are reduced, further indicating that cells may redistribute metabolic resources by reducing the synthesis of aromatic amino acids. These downregulated pathways, in stark contrast to the previously upregulated mineral absorption, peroxisome, and tryptophan metabolism pathways, together form a global metabolic reprogramming network for cells to cope with alkaline stress, achieving environmental adaptation by precisely regulating the expression levels of different functional modules.

### 3.9 RT-qPCR validation of differentially expressed genes

**Figure 9:**
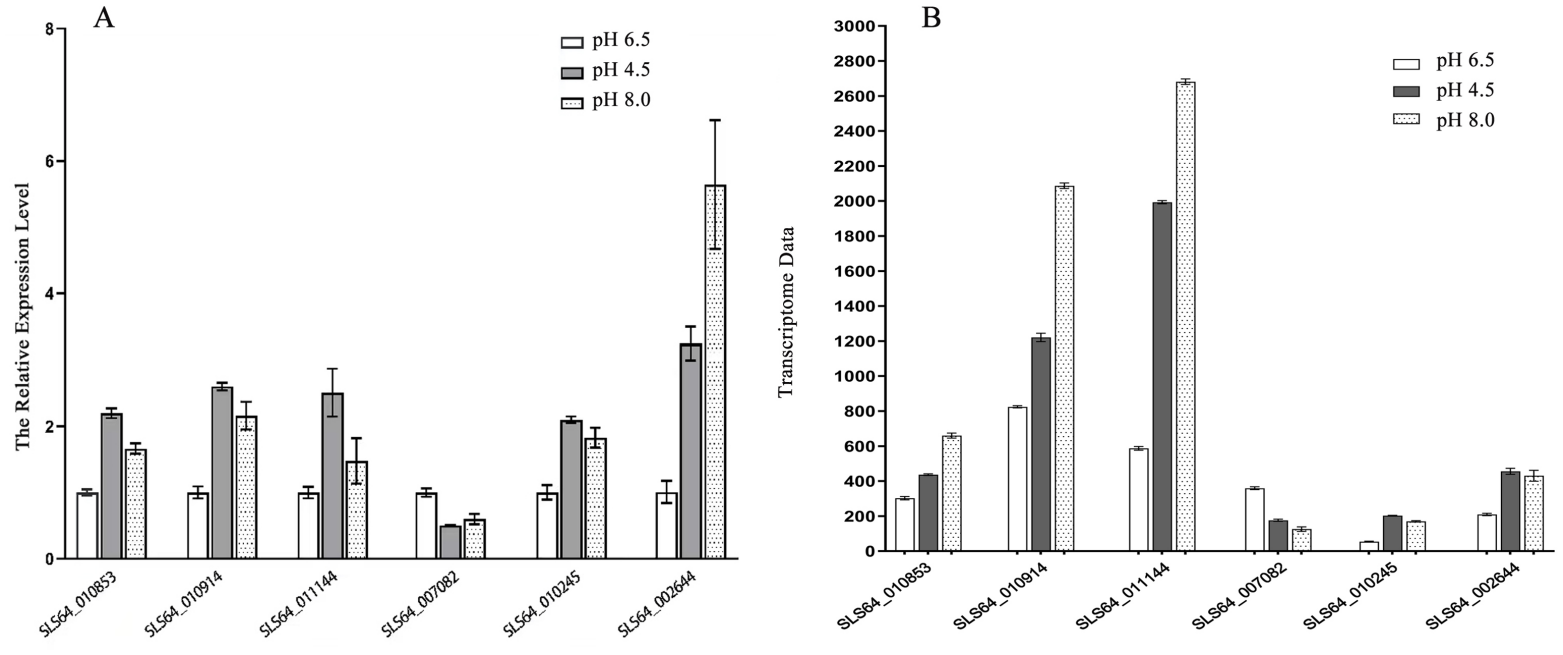
RT-qPCR verification of target gene expression levels. **(A)** RT-qPCR detection of six target gene expression levels in *Diaporthe eres* under acidic or alkaline stress. **(B)** Target gene expression levels in transcriptome data.

To verify the reliability of the RNA-seq data, six genes (SLS64_010853, SLS64_010914, SLS64_011144, SLS64_010245, SLS64_007082, and SLS64_002644) were selected from the transcriptome sequencing results for RT-qPCR validation. The detection results are shown in Figure 9A. The expression trends of SLS64_010853, SLS64_010914, SLS64_011144, SLS64_010245, and SLS64_002644 genes were all upregulated under acidic and alkaline conditions, while SLS64_007082 was downregulated in both conditions (Figure 9A). The above results were consistent with the transcriptome data (Figure 9B), verifying the reliability of the RNA-seq results.

## 4 Discussion and conclusion

Current research on *P. emblica* diseases has mainly focused on fruit-related infections. However, root rot disease in *P. emblica* has not been previously reported. This study is the first to report the identification of root rot in *P. emblica* caused by *Diaporthe eres*, providing novel insights into disease management and cultivation practices for this species. Therefore, based on the investigation results of the first discovery of root rot disease on *Phyllanthus emblica*, this study conducted pathogen identification, investigated the bionomics of *Diaporthe eres*, and evaluated the *in vitro* toxicity of different fungicides.

Diaporthe is a significant plant pathogen with a broad host range, affecting various crops, trees, and ornamental plants (Yang et al., 2021). Reported hosts include species from families such as Rutaceae, Theaceae, Leguminosae, Rosaceae, Sapindaceae, and Vitaceae, including *Pyrus* spp. (Xu et al., 2018), *Citrus reticulata* (Huang et al., 2015), *Juglans regia* (Liu et al., 2020), *Mangifera indica* (Lim et al., 2019), *Vitis vinifera* (Dissanayake et al., 2015), and *Camellia oleifera* (Yang et al., 2023). Diaporthe infections can cause diverse plant diseases, including root rot, fruit rot, top blight, ulcer disease, leaf spot disease, leaf blight, rot, and wilt disease (Gomes et al., 2013). This pathogen is known to coexist with its host and become pathogenic under conditions of reduced plant immunity or metabolic imbalance (Díaz et al., 2017; Wang X. H. et al., 2022; Wang Y. et al., 2022).

This study identified *Diaporthe eres* as the causative agent of *P. emblica* root rot. Glucose and sodium nitrate was the optimal carbon and nitrogen sources for mycelial growth of *Diaporthe eres*, respectively. On the other hand, a previous study by Yan et al. (2015) on *Diaporthe eres* causing blueberry bud blight in Shenyang reported soluble starch and peptone as the optimal carbon and nitrogen sources. These differences may be attributed to variations in fungal strains from different geographical regions and host plants. Future research should further explore the adaptability of the *P. emblica* root rot pathogen to different monosaccharides and polysaccharides, as well as organic and inorganic nitrogen sources. Moreover, no sexual spores of *Diaporthe eres* were observed in this study, and further investigations will be conducted to determine the underlying reasons.

*P. emblica* thrives in dry, warm, and hot climates and grows well in fertile, neutral, and acidic soils but poorly in low-lying, waterlogged areas and calcareous soils. Considering the optimal growth conditions of *Diaporthe eres* identified in this study, *P. emblica* should be cultivated in neutral soil environments. If the soil is slightly acidic, its pH can be adjusted by incorporating plant ash, which not only supplements nutrients but also provides certain preventive effects against the disease. Furthermore, the application of alkaline fungicides or growth regulators may help control root rot disease in *P. emblica*. To validate the feasibility and effectiveness of these strategies, field experiments are planned, and necessary adjustments will be made to plant protection strategies based on the results to ensure they align with the environmental and management requirements of *P. emblica* cultivation.

Chemical control has always been an important strategy for managing fruit and vegetable diseases. This study identified Prochloraz as the most effective fungicide against *Diaporthe eres*, with an EC_50_ value of 0.059 mg/L. Prochloraz, a sterol demethylase inhibitor (DMI), may exert its inhibitory effect due to the sensitivity of *Diaporthe eres* to this class of fungicides, consistent with findings by Yan et al. (2015), Tao et al. (2020), Wang X. H. et al. (2022), and Wang Y. et al. (2022). In actual production, rotating fungicides with different mechanisms of action is essential for delaying resistance development and extending their efficacy in disease management.

The research found that the acid–base environment significantly affects the transcriptional level of *Diaporthe eres*. Under acidic conditions, there were a total of 1,413 differentially expressed genes (DEGs), including 803 up-regulated genes and 610 down-regulated genes. Under alkaline conditions, there were 933 DEGs, including 452 up-regulated genes and 481 down-regulated genes. The reliability of the RNA-seq results was verified by RT-qPCR detection of the expression levels of target genes. The results of this study indicate that when *Diaporthe eres* is subjected to acid stress, it maintains osmotic balance and redox homeostasis by significantly activating amino acid metabolism, while reducing the energy consumption of protein synthesis and RNA biosynthesis by down-regulating ribosome and transcription-related genes (Clark et al., 2012). Under alkaline conditions, Diaporthe eres up-regulates mineral absorption and peroxisome function, enhances copper ion uptake, fatty acid *β*-oxidation, and reactive oxygen species (ROS) scavenging capacity and cooperates with tryptophan metabolism to support NAD^+^ compensation and redox balance. At the same time, the general down-regulation of ribosome and RNA polymerase complex genes, as well as the suppression of aromatic amino acid biosynthesis, reflects a global adaptation pattern of “increasing ions/antioxidants and reducing proteins/transcription.” This study, through the research on genes with expression changes, contribute to a better understanding of the molecular mechanism of *Diaporthe eres* infection in the host in the future.

## Data Availability

The datasets presented in this study can be found in online repositories. The names of the repository and accession numbers are as follows: https://www.ncbi.nlm.nih.gov/, PRJNA1284586, PV170938, PV177466, and PV177465.

## Footnotes

^1^ https://www.ncbi.nlm.nih.gov/datasets/genome/GCA_022570775.2/
