# Supplementary material for: Research on the pathogen causing root rot on Phyllanthus emblica from the perspectives of identification, bionomics, fungicide sensitivity assay, and transcriptome analysis under different pH stress
Source: Front Microbiol. 2025 Jul 31;16:1612979. doi: 10.3389/fmicb.2025.1612979 (PMC12350293; doi:10.3389/fmicb.2025.1612979)
Supplement: Supplementary file 1 [file Data_Sheet_1.docx]

Supplementary Material

# Supplementary Tables

**Table S1.** Primers for amplifying gene fragments in multi-locus sequence analysis.

| Gene | Primer | Primer Sequence(5′-3′) | Annealing temperature/℃ |
| --- | --- | --- | --- |
|  |  |  |  |
| ITS | ITS1 | TCCGTAGGTGAACCTGCGG | 53 |
|  | ITS4 | TCCTCCGCTTATTGATATGC |  |
| *EF1-α* | EF1-728F | CATCGAGAAGTTCGAGAAGG | 54 |
|  | EF1-986R | TACTTGAAGGAACCCTTACC |  |
| *TUB2* | Bt2a | GGTAACCAAATCGGTGCTGCTTTC | 59 |
|  | Bt2b | ACCCTCAGTGTAGTGACCCTTGGC |  |

**Table S2.** Detailed information on the six fungicides involved in this study

| Fungicide (Purity %) | Manufacturer |
| --- | --- |
| Tebuconazole (97.5%) | Hubei Supuer Chemical Co., Ltd., Wuhan, China |
| Difenoconazole (95%) | Hangzhou Panfeng Agricultural Science and Technology Co., Ltd., Hangzhou, China |
| Chlorothalonil (98.0%) | Shaanxi Xinye Technology Development Co., Ltd., Xian, China |
| Prochloraz (95.0%) | Changzhou Tianzhe Chemical Co., Ltd, Changzhou, China |
| Carbendazim (95.0%) | Wuhan Pushida Biotechnology Co., Ltd., Wuhan, China |
| Azoxystrobin (98.5%) | Zhangzhou Jiayi Biotechnology Co., Ltd.Zhangzhou, China |

**Table S3**. Primer sequences for quantitative real-time PCR

| Genes | Primer name | Primer Sequences(5'→3’) |
| --- | --- | --- |
| *SLS64_010853* | *SLS64_010853*－F | CTTGAGCACCTCGTCCTTGT |
|  | *SLS64_010853*－R | TCGACAACGATGCTGAACCA |
| *SLS64_010914* | *SLS64_010853*－F | GGATCCTGCACCAACTCCTC |
|  | *SLS64_010853*－R | TCAACGTAGTCCTTGCCGTC |
| *SLS64_011144* | *SLS64_010853*－F | TCCTTGCCGAATGGTAACCC |
|  | *SLS64_010853*－R | TTTTGCCAAACTGCCCGAAG |
| *SLS64_010245* | *SLS64_010853*－F | GCAGCTGACCCCGAACTAAA |
|  | *SLS64_010853*－R | GTGAAGCACGTTGAACCAGC |
| *SLS64_007082* | *SLS64_010853*－F | GGTCTACCAGGCTCTCATGC |
|  | *SLS64_010853*－R | GGTCCTCCAGGTTGTACAGC |
| *SLS64_002644* | *SLS64_010853*－F | TTGGGCTGGTTTGGCAGTAA |
|  | *SLS64_010853*－R | TCTTTCGACACCAAGGCCTC |
| *Tubulin* | *Tubulin*－F | CCCCGTCTGCACTTCTTCAT |
|  | *Tubulin*－R | TCTTGGGGTCGAACATCTGC |

**Table S4**. The toxicity of six chemical fungicides on the growth of colonies of *Diaporthe eres* YX-1.

| Fungicide | concentrations  (μg/mL) | Colony diameter with fungicide containing (mm) | Control (mm) | Inhibition rates (%) |
| --- | --- | --- | --- | --- |
|  |  |  |  |  |
| Tebuconazole (97.3%) | 0.1 | 52.5±1.53a | 55.4±2.04a | 5.23 |
|  | 0.2 | 49.0±1.15b |  | 11.55 |
|  | 0.4 | 42.6±1.15c |  | 23.10 |
|  | 0.8 | 32.9±1.91d |  | 40.61 |
|  | 1.6 | 19.1±1.50e |  | 65.52 |
|  | 3.2 | 11.3±1.20f |  | 79.60 |
|  | 6.4 | 7.0±0.82g |  | 87.36 |
| Difenoconazole (95%) | 0.01 | 50.3±1.31b | 56.3±1.61a | 10.66 |
|  | 0.02 | 43.7±1.05c |  | 22.38 |
|  | 0.05 | 39.3±1.51d |  | 30.20 |
|  | 0.1 | 32.5±0.90e |  | 42.27 |
|  | 0.2 | 23.0±1.35f |  | 59.15 |
|  | 0.5 | 13.8±0.96g |  | 75.49 |
|  | 1 | 9.2±0.75h |  | 83.66 |
| Prochloraz (95%) | 0.005 | 51.9±1.51b | 54.8±1.75a | 5.29 |
|  | 0.01 | 45.8±1.15c |  | 16.42 |
|  | 0.02 | 39.3±1.11d |  | 28.28 |
|  | 0.05 | 31.1±1.32e |  | 43.25 |
|  | 0.1 | 21.2±0.98f |  | 61.31 |
|  | 0.2 | 11.1±0.89g |  | 79.74 |
|  | 0.5 | 6.2±0.62h |  | 88.68 |
| Carbendazim (95%) | 0.05 | 51.6±1.15a | 54.4±1.03a | 5.15 |
|  | 0.1 | 46.5±1.90b |  | 14.52 |
|  | 0.2 | 36.9±1.04c |  | 32.17 |
|  | 0.5 | 30.6±1.20d |  | 43.75 |
|  | 1 | 22.6±1.39e |  | 58.46 |
|  | 2 | 11.8±0.95f |  | 78.31 |
|  | 5 | 7.4±0.66g |  | 86.40 |
| Azoxystrobin (98.5%) | 2 | 53.0±1.15b | 57.8±1.15a | 8.30 |
|  | 5 | 47.1±1.15c |  | 18.51 |
|  | 10 | 42.1±1.15d |  | 27.16 |
|  | 20 | 35.4±1.15e |  | 38.75 |
|  | 50 | 27±1.15f |  | 53.29 |
|  | 100 | 15.1±1.15g |  | 73.88 |
|  | 200 | 8.6±1.15h |  | 85.12 |
| Chlorothalonil (98%) | 0.5 | 52.2±1.27b | 56.8±0.96a | 8.10 |
|  | 1 | 47.0±1.40c |  | 17.25 |
|  | 2 | 38.9±1.47d |  | 31.51 |
|  | 5 | 28.7±0.76e |  | 49.47 |
|  | 10 | 21.7±1.08f |  | 61.79 |
|  | 20 | 13.1±1.53g |  | 76.94 |
|  | 50 | 7.8±0.56h |  | 86.27 |

Table S5. In vitro toxicity of six fungicides to *Diaporthe eres* YX-1.

| Fungicide | Toxicity regression equations | R correlation coefficient | EC_50_ (μg/mL) | 95% CI (ug/mL) |
| --- | --- | --- | --- | --- |
|  |  |  |  |  |
| Tebuconazole (97.3%) | y=1.620x-0.043 | 0.997 | 1.063 | 0.910-1.246 |
|  |  |  |  |  |
| Difenoconazole (95%) | y=1.096x+0.978 | 0.996 | 0.128 | 0.104-0.160 |
|  |  |  |  |  |
| Prochloraz (95%) | y=1.372x+1.689 | 0.995 | 0.059 | 0.049-0.071 |
|  |  |  |  |  |
| Carbendazim (95%) | y=1.313x+0.277 | 0.991 | 0.616 | 0.511-0.745 |
|  |  |  |  |  |
| Azoxystrobin (98.5%) | y=1.185x-1.787 | 0.995 | 32.226 | 26.340-39.858 |
|  |  |  |  |  |
| Chlorothalonil (98%) | y=1.239x-0.921 | 0.997 | 5.543 | 4.564-6.758 |

Table S6. Statistics of the transcriptome data.

| Sample | Raw reads | Clean reads | Mapped reads | Q20 (%) | Q30 (%) | GC content |
| --- | --- | --- | --- | --- | --- | --- |
| A_1_ (pH=4.5) | 9891620 | 9881832 | 8391148 (84.91%) | 99.38 | 97.70 | 57.72 |
| A_2_ (pH=4.5) | 10635782 | 10524062 | 8911222 (84.67%) | 99.17 | 97.08 | 57.67 |
| A_3_ (pH=4.5) | 8705424 | 8602144 | 7554676 (87.82%) | 98.79 | 95.90 | 57.70 |
| B_1_ (pH=6.5) | 11746784 | 11589394 | 9827414 (84.79%) | 99.36 | 97.65 | 57.47 |
| B_2_ (pH=6.5) | 11784653 | 11652356 | 9851101 (84.54%) | 99.09 | 96.83 | 57.45 |
| B_3_ (pH=6.5) | 9945124 | 9833966 | 8976512 (91.28%) | 98.76 | 95.92 | 57.52 |
| C_1_ (pH=8.0) | 9256487 | 9137616 | 7683837 (84.09%) | 99.29 | 97.47 | 57.36 |
| C_2_ (pH=8.0) | 7465987 | 7347954 | 6867010 (93.45%) | 99.31 | 97.50 | 57.66 |
| C_3_ (pH=8.0) | 10478453 | 10210152 | 8598222 (84.21%) | 99.27 | 97.42 | 57.24 |
